# Racial/Ethnic and social class differences in preventive care practices among persons with diabetes

**DOI:** 10.1186/1471-2458-6-259

**Published:** 2006-10-19

**Authors:** Carol R Williams Oladele, Elizabeth Barnett

**Affiliations:** 1 University of South Florida, College of Public Health, Department of Epidemiology and Biostatistics, 13201 Bruce B. Downs Blvd. MDC 56, Tampa FL 33612 USA

## Abstract

**Background:**

Diabetes is the sixth leading cause of death in the United States. Persons with diabetes are at increased risk for serious complications including CVD, stroke, retinopathy, amputation, and nephropathy. Minorities have the highest incidence and prevalence of diabetes and related complications compared to other racial groups. Preventive care practices such as smoking cessation, eye examinations, feet examinations, and yearly checkups can prevent or delay the incidence and progression of diabetes related complications. The purpose of this study was to examine racial/ethnic differences in diabetes preventive care practices by several socio-demographic characteristics including social class.

**Methods:**

Data from the Behavioral Risk Factor Surveillance Survey for 1998–2001 were used for analyses. The study population consisted of persons who indicated having diabetes on the BRFSS, 35 yrs and older, and Non-Hispanic Black, non-Hispanic White, or Hispanic persons. Logistic regression was used in analyses.

**Results:**

Contrary to our hypotheses, Blacks and Hispanics engaged in preventive care more frequently than Whites. Whites were less likely to have seen a doctor in the previous year, less likely to have had a foot exam, more likely to smoke, and less likely to have attempted smoking cessation. Persons of lower social class were at greatest risk for not receiving preventive care regardless of race/ethnicity. Persons with no health care coverage were twice as likely to have not visited the doctor in the previous year and twice as likely to have not had an eye exam, 1.5 times more likely to have not had a foot exam or attempted smoking cessation.

**Conclusion:**

This study showed that persons of lower social class and persons with no health insurance are at greatest risk for not receiving preventive services.

## Background

Diabetes is the sixth leading cause of death in the United States [1]. It is a major cause of premature mortality and morbidity and poses an enormous economic burden. Direct medical and indirect expenditures attributable to diabetes in 2002 alone were estimated at 132 billion dollars [2,3]. This disease affects an estimated 18.2 million persons of whom 5.2 million are unaware of having the disease and over 1 million new cases are diagnosed yearly [1,3]. Heart disease is the leading cause of death among persons with diabetes with mortality rates two to four times higher than those without diabetes [1,4]. Preventive care practices such as diet, exercise, smoking cessation, eye examinations, foot examinations, and yearly checkups can prevent or delay the incidence and progression of diabetes related complications [4-6]. However, the frequency of preventive care practices is often lower than recommended which results in further progression of disease and mortality [4,5,7-9].

The burden of diabetes is greater among minority populations [10,11]. An estimated 8.4% of Whites, 11.4% of Blacks, and 8.2% of Hispanics 20 years or older have diabetes [1]. The increasing incidence and prevalence of diabetes is seen at disproportionate levels among ethnic minorities. During the period 1980–1994 the age-adjusted prevalence of diabetes increased 33% for Blacks compared to an 11% increase among Whites [10].

Few studies have simultaneously investigated racial/ethnic and social class differences in care practices associated with preventive care behaviors among persons with diabetes. Results from the 1995 and 2001 Behavioral Risk Factor Surveillance System (BRFSS) show variation in preventive-care practices among Whites, Hispanics, and Blacks [4,5]. In contrast, other studies have found no racial/ethnic differences in preventive care practices for diabetes [8,12]. Findings in the literature are somewhat inconsistent and limited in their method of exploring factors related to care practices. The present study utilizes recent BRFSS data available to examine racial/ethnic disparities in recommended preventive practices for diabetes, refines past measures of social class with the inclusion of labor force status in addition to educational attainment, and makes use of logistic regression in analyses instead of previously used weighted percentages.

## Methods

### Data

The Behavioral Risk Factor Surveillance System (BRFSS) is a state based random-digit-dialing telephone survey of non-institutionalized individuals (e.g. nursing homes, prisons) 18 years or older [4,13]. The core questions in the BRFSS cover topics such as health status, routine checkup, diabetes, smoking, pregnancy, women's health, HIV/AIDS, and socio-demographics [4,13,14]. This study was conducted under the Adverse Heart Disease Trends Project and approved by the Institutional Review Board at the University of South Florida.

### Study Population

The study population consisted of persons who met the following criteria: (A) Residents of one of the 37 states who participated in the diabetes module during 1998–2001; (B) Non-Hispanic Black, non-Hispanic White, and Hispanic persons; (C) 35 years and older; (D) Answered "yes" to the following question: "Has a doctor ever told you that you have diabetes?" Women who indicated having gestational diabetes were not included. The total number of persons included in the study population was 23,434.

The BRFSS computed index for race/ethnicity was used for analyses. This index categorized self-reported race/ethnicity by Hispanic origin. Analyses were restricted to Non-Hispanic Blacks, Non-Hispanic Whites, and Hispanic respondents. Other racial/ethnic groups were not included because of very small numbers of BRFSS respondents. Persons with type-1 and type-2 diabetes were combined in analyses since 90–95 percent of diabetes cases are type 2 and the preventive practices examined in the current study are the same for both groups. The age range for analyses were limited to those 35 years and older since this age range encompasses the greater proportion of diabetes cases. This age range also allows examination of preventive care practices associated with many end stage diabetes outcomes.

### Preventive Care Measures

The diabetes preventive practices chosen for analysis were annual doctor visit for diabetes, annual eye examination, annual foot examination, and attempted smoking cessation. These measures were all assessed in the diabetes module of the BRFSS. Only respondents who reported a diagnosis of diabetes were asked module questions. Preventive care practices such as hemoglobin A1C, physical activity, and diet were not assessed due to high missing or incomparability across study years. Questions reflect recommendations given by the American Diabetes Association (Table 1).

Responses of "Don't Know", "Refused," or "Not Sure" were coded as missing for smoking, attempted smoking cessation, doctor visit, and foot examination. Similar responses to eye examination were coded as "No" since respondents would most likely remember having their eyes dilated if indeed they did have an exam. Respondents who refused to answer this question were coded as missing.

### Demographic Measures

Socio-demographic characteristics hypothesized to influence performance of diabetes care practices were also examined. These measures included race, marital status, education, age, employment, and gender. Persons interviewed for the BRFSS were asked, "What is your race," and given a list of responses from which to choose. Respondents to the marital status measure were categorized as "Married," "Widowed," "Separated," "Divorced," "Never Been Married," and "Member of an unmarried couple." Respondents who answered, "Never been married" or "Member of an unmarried couple" on the marital status measure were collapsed into one category called "Never been married." Those who responded as having been "Separated" were combined with "Divorced." Education level categories were 0–8 years, 9–11 years, high school graduate, some college, and college and beyond. Respondents indicated the highest level of education attained. Subjects with at least a kindergarten education and those with 1 to 8 years were combined. Employment was evaluated based on respondents' current employment status.

A social class index was created using employment status and education to assess the influence of labor force participation. This index is based on Weberian sociology that focuses on the role of individual social interactions that determine a person's opportunities in life [15]. A summary of the index is illustrated in Table 2. Other descriptive information included age at first diagnosis and health plan. Health plan status was categorized as having or not having a health care coverage plan. Responses of "Don't Know", "Refused," or "Not Sure" were coded as missing for all demographic variables.

### Statistical and Analytical Methods

Given that minorities have greater and more severe end-stage diabetes complications, we hypothesized that (A) Blacks and Hispanics would receive less preventive care after controlling for independent variables; (B) Persons of lower social class would receive less preventive care; and (C) Women would engage in care practices more than men. Each preventive care practice was examined individually for the entire study population. Attempted smoking cessation was only assessed for persons who indicated that they were current smokers. Duration of diabetes and insulin measures was included since levels of preventive care and perceived severity of disease correlate with insulin use.

Logistic regression was performed to estimate odds ratios with 95% CI for the independent effect of each variable. In all analyses social class index 1 and male gender were referent categories. The age referent category was different for each model since the lowest risk group for each outcome varied.

To measure the unadjusted effect of race/ethnicity on each care practice, a crude model was examined. Results were compared with another model, which included social class index, gender, health plan status, insulin use, duration of diabetes, and age. Marital status was excluded after initial analyses showed no association. Two-way interactions were also examined for race/ethnicity and social class index. Analyses were performed using the SAS statistical software package [16]. The odds of not having received preventive care were modeled in regression analyses.

## Results

Table 3 summarizes the sociodemographic characteristics of the study population. The study population was 81 percent White, 11 percent Black, and 8 percent Hispanic. Eight percent of persons did not have any form of health care coverage. Most study participants (50%) fell in social class IV who were those who are out of the labor force (homemakers, students, retirees). Fifty-eight percent of the population was women. Approximately 51 percent of the study population was between the ages of 55–74 while 10 percent of the population was 35–44 and 20 percent 45–54. Twenty-nine percent of persons were insulin users and the majority of participants were married (50%).

### Race/Ethnicity

Blacks and Hispanics engaged in preventive care practices more frequently than Whites and race/ethnicity was not a strong predictor for any of the care practices examined. (Table 4) The crude model showed weak but significant results for race/ethnicity. Compared to Whites, Blacks were less likely to have not seen a doctor in the previous year, less likely to have not had a foot exam, and less likely to have not attempted smoking cessation. However, this model did show Blacks were more likely to smoke (OR = 1.2). Hispanics were more likely to have not had an eye exam (OR = 1.2) and were more likely to have attempted cessation compared to Whites (OR = .6).

The adjusted model showed similar results for Blacks. After adjustment however, Blacks were less likely to smoke compared to Whites. The latter finding was not statistically significant. Previous findings for Hispanics were no longer significant after adjustment except for attempted cessation. Hispanics were still more likely to have attempted smoking cessation compared to Whites. Adjusted results also showed Hispanics were less likely to smoke. An interaction term for race/ethnicity and social class was also added to the model but the terms were not statistically significant.

### Social Class

Though overall findings for race/ethnicity were weak, those for social class showed strong patterns. This was especially true for eye examination and smoking. A positive gradient was observed for social class 1 through 3 for eye exam. Persons who were employed and had less than a high school education were more likely to have not had an eye examination compared to those employed and college educated. Persons out of the labor force (retirees, students, homemakers) had a 20 percent increased odds for not having had an eye exam while unemployed persons had a 30 percent increased odds. Those unable to work had odds similar to those of persons in social class 2 (employed & high school graduate) with a 40 percent increased odds of not receiving a foot examination compared to those in social class 1 (college educated and employed).

The strongest social class findings were those for smoking. Results for smoking showed a strong positive gradient for social class 1 to 3. Compared to persons in social class 1 (employed & college educated) persons in social class 2 and 3 had two times and three times the odds of smoking respectively. Persons out of the labor force and those unemployed had twice the odds of smoking compared to those in social class 1. Results for the unemployed had strong results that mirrored those for persons in social class three (employed with less than a high school education) having close to three times the odds of smoking (OR = 2.8). There was a small gradient observed for attempted smoking cessation. However, the associations were weak and not statistically significant.

Findings for yearly doctor visit showed a weak association with social class 1 to 5 and those unable to work. Similar results were observed for foot examination except for the finding for persons in social class 3. Results showed that persons in social class 3 (those employed with less than a high school education) were almost twice as likely to have not had a foot examination compared to those employed and college educated. Other results for foot examination were weak and not statistically significant.

### Health Care Coverage

Results for health coverage were statistically significant and congruent with previous findings [9,17]. In fact, health care coverage was strongly associated with all the preventive care practices. Persons with no health care coverage were twice as likely to have not visited the doctor in the previous year and twice as likely to have not had an eye exam, 1.5 times more likely to have not had a foot exam or attempted cessation. Persons without health care coverage also had a 30 percent increased prevalence of smoking.

### Gender and Age

Women were at least risk for not having had many of the preventive care practices. Results for doctor visit, eye examination, and smoking showed women had favorable risk compared to men. However, only the results for smoking were statistically significant. Women were found to be at greater risk for not having had a foot examination (OR = 1.2). This finding was statistically significant. Results for age (table 4) show persons 35–44 are more likely to have not received preventive care. The exception however, was smoking cessation. This age group was more likely to have attempted cessation compared to older age groups.

### Insulin Use and Duration of Diabetes

Non-insulin dependent persons were at greater risk for not getting preventive care. Persons who did not use insulin were 3.4 times more likely to have not visited the doctor in the past year. Results for eye examination showed that non-insulin dependent persons were almost twice as likely to have not received an eye examination compared to persons who used insulin. Non-insulin dependent persons were also more likely to not have had a foot examination (OR = 1.9). Insulin use was not a strong predictor for smoking or attempted smoking cessation. Odds ratios were 1.1 and 1.2 respectively and neither was statistically significant.

Duration of diabetes showed no relationship with any of the preventive care practices examined.

## Discussion

The findings presented in this paper confound past theories and explanations for disparities seen among persons with diabetes. It is well established that ethnic differences in end-stage outcomes for diabetic persons exist. However, our results show Black and Hispanic persons with diabetes have higher levels of preventive care, which is not congruent with observed end-stage diabetes outcomes [18-21]. The question still remains of why disparities in end-stage outcomes persist despite the higher levels of preventive care. One explanation is that Blacks and Hispanics present with more severe disease and a greater number of co-morbidities. Therefore, providers may be more likely to adhere to the recommended care guidelines examined here. Another explanation is that there may be lower adherence to preventive practices not measured here among Black and Hispanic persons. There may also be an economic barrier present in implementing suggested changes after a physicians visit.

Differences in care practices between private and public insurance may also explain results. For example, if it were the case that government sponsored plans facilitated behavioral type preventive care practices (e.g.. eye exam, food exam) easier than private insurers, we may observe the current results. Harris (1999) found that a greater proportion of Whites (91%) and Blacks (89%) had health insurance coverage compared to Hispanics (66%). Of those having health care coverage, a disproportionate number of Blacks and Hispanics had some form of government-sponsored health insurance compared to Whites. Only 56% of Blacks and 45% of Hispanics had private insurance [10]. A recent study conducted in Puerto Rico found that the use of laboratory services like glucose and glycosilated hemoglobin tests was greater in patients who had private insurance [22]. This finding supports the hypothesis that differences in care practices do exist between the private and public insurance sector. This would also support findings from this and other studies that Blacks and Hispanics perform lower levels of glycemic control activities and lab checks in comparison to self-care practices where performance levels are higher compared to Whites [8,17].

Others have found contrasting results showing that medical/laboratory checks are delivered more frequently than behavioral activities and that Blacks maintain a more favorable laboratory profile [23,24]. If the hypothesis is true that government sponsored health care promotes self-care activities more than medical/laboratory type practices, and given that a great number of Black persons with diabetes have some form of government health care, then previous findings make sense. Glasgow and Strycker (2000) found self-management activities were recommended less frequently than medical/laboratory checks for persons with diabetes in two private health care systems. This may point towards differences in recommendations and practice between private and government sponsored insurers.

The American College of Emergency Physicians has shown that some 19 and 33 percent of Blacks and Hispanics are uninsured [25]. A study utilizing 1999 BRFSS data also found that more Blacks were uninsured compared to Hispanics and Whites and reported cost as a barrier to visiting a doctor [26]. The cost barrier may further lead to under use of services and essential medication that impact the health of persons with diabetes [27].

The strong patterns observed for social class are not surprising given the demonstrated relationship between social class and chronic illnesses [28-30]. Results in the current study showed a strong association between social class and preventive care practices above the influence of race/ethnicity. This was not too surprising given that race is such a powerful determinant of individuals position within the class structure of society [31]. Lower social class has also been associated with increased diabetes complications [32,33]. Although we controlled for health plan status in analyses, a residual effect is likely between social class and health insurance since they are so strongly associated and health insurance status differs by level of social class. Persons in higher social class are more likely to be insured compared to persons in lower levels, even though the rising cost of care and employer cutbacks on insurance is creating a growing middle class that is uninsured.

The findings in this paper are subject to a few limitations. First, persons with diabetes may have been missed with the question "Has a doctor ever told you that you have diabetes." Interviewed persons who do not have access to health care services may have answered "no" while they may unknowingly have diabetes. This potential bias may have resulted in obtaining measures of association that are weaker than the true measure since persons who lack health care access are more likely to be diagnosed at more severe stages and not receive preventive care. Second, the social class index used here may not fully capture social class due to the inherent limitations of the two proxy measures used (education and employment status) in capturing such a complex concept. Though there is no agreed upon way to measure social class, education and employment have been used extensively in previous work as proxy measures [34-36]. The combination of the two variables, which was used to create the social class index, has not been validated; however findings using this measure are consistent with previous findings on social class in the literature [34,36]. Another limitation is that analyses included only non-institutionalized persons, which limit populations to which results can be generalized. Recall bias is another limitation as persons were asked to remember past preventive care practices. The extent to which the bias inherent in this telephone survey affects results is unknown. Percent missing for key variables was as follows: yearly doctor visit (4%), foot examination (10%), eye examination (0.3%), smoking (0.2%), health insurance coverage (0.4%), and employment (0.1%). Subsequently, results may not reflect true measure of association between race/ethnicity and preventive care practices, especially if missing is systematic. A final limitation is the way in which race/ethnicity was determined. Respondents were given a list of responses to choose which may have omitted responses for some race/ethnic groups.

The BRFSS is a telephone survey that does not reach persons without phone service. Persons missed by this survey method are more likely to be poor and minority [37]. Thus, results may be an overestimate of preventive care practices among minorities since high-risk persons may be excluded. Future studies should include these high-risk populations who may have been excluded in the present study.

## Conclusion

These results suggestthat recenthealth education and promotion efforts targetedtowardminority populations have been effective.However, serious complications from diabetes still continue to be highest among minorities. Future research should examine theinfluenceof type of insuranceon diabetes patients' access to laboratory preventive care practices. Finally, prevention efforts should be geared toward earlier screening anddiagnosis and better access to preventive and medical services for bothminorities and whites.

## Competing interests

The author(s) declare that they have no competing interests.

## Authors' contributions

CWO contributed to the conception, design, analysis, and drafting of the manuscript. EB contributed to the design, analysis, and preparation of the manuscript. Both authors contributed to editing the manuscript.

## Pre-publication history

The pre-publication history for this paper can be accessed here:



## Figures and Tables

**Table 1:** Preventive Care Practice Questions

1. About how many times in the last year have you seen a doctor, nurse of other health professional for your diabetes?
2. About how many times in the last year has a health professional checked your feet for any sores or irritations?
3. When was the last time you had an eye exam in which the pupils were dilated? This would have made you temporarily sensitive to bright light.
4. During the past 12 months, have you quit smoking for 1 day or longer?

**Table 2:** Social Class Index

Level	Indicators	
	Employment Status	Educational Attainment

SCL 1	Employed for Wages or Self-Employed	College Graduate
SCL 2	Employed for Wages or Self-Employed	High School Graduate
SCL 3	Employed for Wages or Self-Employed	< High School Graduate
SCL 4	Out of Labor Force (Students, Retirees, Homemakers	All Levels
SCL 5	Unemployed	All Levels
Unable to Work	Unable to Work	All Levels

**Table 3:** Characteristics of Study Population, 1998–2001

Characteristic	Total	Black	White	Hispanic
N(%)				

**Race**				
Black	2485(11)			
White	18947(81)			
Hispanic	2002(8)			

**No Insurance Coverage**	1940(8.3)	315(12.7)	1250(6.6)	375(18.8)

Social Class*				
SCL I	2048(8.8)	187(7.6)	1764(9.4)	97(4.9)
SCL II	4847(20.8)	561(22.7)	3893(20.6)	393(19.7)
SCL III	842(3.6)	139(5.6)	487(2.6)	216(10.8)
SCL IV	11641(49.9)	879(35.6)	9952(52.8)	810(40.7)
SCL V	639(2.7)	112(4.5)	440(2.3)	87(4.4)
Unable to Work	3290(14.1)	589(23.9)	2313(12.3)	388(19.5)

**Gender**				
Women	13643(58.2)	1646(66.2)	10799(57.0)	1198(59.8)

**Age**				
35–44	2359(10.1)	328(13.2)	1702(9)	329(16.4)
45–54	4731(20.2)	649(26.1)	3573(18.9)	509(25.4)
55–64	5839(24.9)	694(27.9)	4613(24.4)	532(26.6)
65–74	6208(26.5)	553(22.3)	5228(27.5)	427(21.3)
75–84	3710(15.8)	231(9.3)	3295(17.4)	184(9.2)
85+	587(2.5)	30(1.2)	536(2.8)	21(1.1)

**Insulin Use**	6815(29.1)	922(37.2)	5324(28.1)	569(28.5)

**Median Age at Diagnosis**	51	47	53	47

**Marital Status**				
Married	11784(50.4)	813(32.9)	9913(52.4)	1058(52.9)
Divorced/Separated	4266(18.2)	730(29.6)	3110(16.4)	426(21.3)
Widowed	5515(23.6)	587(23.8)	4590(24.3)	338(16.9)
Single	1820(7.8)	340(13.8)	1303(6.9)	177(8.9)

*SCL I-employed & college educated; SCL II-employed & high school graduate;

SCL III-employed & less than high school ; SCL IV-out of labor force & all levels of education; SCL V-unemployed & all levels of education.

**Table 4:** Predictors of Diabetes Preventive Care Practices/Blacks, Whites, and Hispanics 1998–2001

Characteristic	No Yearly Doctor Visit in Past Year	No Eye Examination	No Foot Examination in Past Year	Smoking	No Attempted Smoking Cessation^e^
	OR	OR	OR	OR	OR
	95% CI	95% CI	95% CI	95% CI	95% CI
**Race (crude model)**					
Black	0.5* (0.4–0.6)	1.0 (0.9–1.1)	0.7* (0.6–0.8)	1.2* (1.1–1.4)	0.6* (0.4–0.7)
White	1.0 (1.0–1.0)	1.0 (1.0–1.0)	1.0 (1.0–1.0)	1.0 (1.0–1.0)	1.0 (1.0–1.0)
Hispanic	1.0 (0.9–1.2)	1.2* (1.1–1.3)	1.1 (1.0–1.2)	1.1 (0.9–1.2)	0.6* (0.5–0.8)
**Race (adjusted model)**					
Black	0.6* (0.5–.7)	0.9 (0.8–1.0)	0.7* (0.6–.7)	1.0 (0.9–1.1)	0.6* (0.5–0.7)
White	1.0 (1.0–1.0)	1.0 (1.0–1.0)	1.0 (1.0–1.0)	1.0 (1.0–1.0)	1.0 (1.0–1.0)
Hispanic	0.8 (0.7–1.0)	1.0 (0.9–1.1)	1.0 (0.9–1.1)	0.7* (0.6–0.8)	0.7* (0.5–0.9)
**Health Plan Status**					
No Health Insurance Coverage	2.1* (1.8–2.4)	2.0* (1.8–2.2)	1.5* (1.3–1.7)	1.3* (1.2–1.5)	1.5* (1.2–1.8)
**Social Class**					
SCL I	1.0^d ^(1.0–1.0)	1.0^d ^(1.0–1.0)	1.0^d ^(1.0–1.0)	1.0^d ^(1.0–1.0)	1.0^d ^(1.0–1.0)
SCL II	1.1 (0.9–1.3)	1.4* (1.2–1.5)	1.1 (1.0–1.3)	1.7* (1.5–2.0)	1.2 (0.9–1.6)
SCL III	1.3 (1.0–1.6)	1.7* (1.5–2.1)	1.6* (1.3–1.9)	2.8* (2.3–3.5)	1.3 (0.8–1.9)
SCL IV	0.8 (0.7–1.0)	1.2* (1.1–1.4)	1.1 (1.0–1.3)	1.8* (1.5–2.1)	0.9 (0.7–1.2)
SCL V	1.1 (0.8–1.5)	1.3* (1.1–1.6)	1.2 (0.9–1.4)	2.4* (1.9–3.0)	0.8 (0.5–1.2)
Unable to Work	0.7* (0.6–.9)	1.4* (1.3–1.6)	1.0 (0.9–1.2)	2.8* (2.4–3.3)	0.7 (0.5–1.0)
**Gender†**					
Women	0.9 (0.8–1.0)	0.9 (0.9–1.0)	1.2* (1.1–1.3)	0.9* (0.8–0.9)	1.0 (0.9–1.2)
**Age**					
35–44	1.4* (1.2–1.7)	2.1* (1.8–2.4)	1.2 (1.0–1.3)	7.3* (5.9–8.9)	1.0^b ^(1.0–1.0)
45–54	1.1 (0.9–1.3)	1.6* (1.4–1.8)	1.0 (0.9–1.1)	5.4* (4.5–6.6)	1.1 (0.9–1.4)
55–64	0.9 (0.8–1.1)	1.2* (1.1–1.4)	1.0 (0.9–1.1)	4.0* (3.3–4.8)	1.3 (1.0–1.6)
65–74	1.0^c ^(1.0–1.0)	1.1 (1.0–1.2)	1.0^c ^(1.0–1.0)	2.4* (2.0–2.8)	1.5* (1.1–2.0)
75–84	1.1 (1.0–1.3)	1.0^a ^(1.0–1.0)	1.2* (1.1–1.3)	1.0^a ^(1.0–1.0)	1.6 (1.0–2.4)
85+	1.3 (1.0–1.7)	1.4* (1.1–1.7)	1.3* (1.1–1.6)	0.3* (0.2–0.6)	1.1 (0.2–5.0)
Insulin Use	3.4* (3.0–3.9)	1.6* (1.5–1.7)	1.9* (1.7–2.0)	1.1 (1.0–1.2)	1.2 (1.0–1.4)
Duration of Diabetes	1.0 (1.0–1.0)	1.0 (1.0–1.0)	1.0 (1.0–1.0)	1.0 (1.0–1.0)	1.0 (1.0–1.0)

Total	22543	23391	21119	23343	3152

Probability of not receiving care practice is modeled

* 95% CI excludes 1.0 ^† ^Male Referent

^a ^Age referent 75–84 yrs., ^b ^Age referent 35–44 yrs., ^c ^Age referent 65–74 yrs. ^d ^SCL I used as referent ^e ^Model only includes smokers
